# Neuro(re)development Of Brain Circuitry: Linking Cell Biology to Psychiatric Discoveries

**DOI:** 10.3389/fpsyt.2013.00065

**Published:** 2013-07-08

**Authors:** B. Ian Hutchins

**Affiliations:** ^1^Cellular and Developmental Neurobiology Section, National Institute of Neurological Disorders and StrokeBethesda, MD, USA

A commentary on Identification of risk loci with shared effects on five major psychiatric disorders: a genome-wide analysis by Cross-Disorder Group of the Psychiatric Genomics Consortium, Smoller JW, Craddock N, Kendler K, Lee PH, Neale BM, Nurnberger JI, Ripke S, Santangelo S, Sullivan PF. Lancet (2013) 381(9875):1371–9. doi: 10.1016/S0140-6736(12)62129-1

Neurodevelopmental defects are thought to contribute to psychiatric disorders by disrupting the proper wiring of neural circuits. While these defects may underlie putative developmental disorders like schizophrenia, their contribution to others, like major depressive disorder, are less clear. Genome-wide association studies (GWAS) have identified many genes associated with individual disorders. However, given the diverse etiologies and ages of onset of common psychiatric disorders, one might expect individual gene variants to associate mainly with one or two disorders sharing common mechanisms (e.g., defects in synaptic transmission or disrupted axon connectivity). It was therefore surprising that the Cross-Disorder Group of the Psychiatric Genomics Consortium recently identified mutations in L-type calcium channels common to five psychiatric disorders (1), not all of which are thought to be neurodevelopmental in origin (autism spectrum disorder, attention deficit-hyperactivity disorder, bipolar disorder, major depressive disorder, and schizophrenia).

One possibility raised by these results is that a fundamental mechanism is used by the nervous system throughout early development and into adulthood, that, when disrupted, increases susceptibility to psychiatric disorders in general. If there were such a fundamental mechanism at work, what would it look like? First, it would of course have to operate through L-type channels. Second, it would have to be utilized broadly in the nervous system to account for the pleiotropic effects of these mutations. Third, it would have to operate both during neurodevelopment and in mature circuitry, since the Psychiatric Genomics Consortium (1) implicated these genes in disorders of varied neurodevelopmental contributions.

Basic research from the last decade has identified a common cell biological mechanism that meets these criteria and could potentially explain this source of shared risk for psychiatric disorders: competitive axon remodeling. During competitive axon remodeling, electrical activity drives a local depolarization that leads to L-type voltage gated calcium channel activation and calcium influx localized to a branch or small group of branches (2). These stimulated branches are favored for extension or further branching, and a competitive mechanism is engaged that causes pruning of unstimulated branches from the same axon (2–4). Blocking L-type calcium channels slows competitive axon remodeling, while stimulating these channels accelerates this process (2), fulfilling the first criterion of dependence on L-type channels. An example of this process is shown in Figure 1. This process has been identified in cortical neurons (2), hippocampal neurons (3), and peripheral neurons (4), meeting the second criterion of broad utilization. Finally, imaging of axon dynamics in the adult neocortex has demonstrated that mature axons continue to dynamically extend and retract branches (5, 6). Although at a lower rate than in pre- and post-natal critical periods, this process still occurs at these later, post-developmental stages of life. In addition to the large-scale axon remodeling in development, the capacity for experience-dependent, competitive axon remodeling is preserved through adulthood in primates as well (7, 8). This shows that axon remodeling is not limited to developmental windows, but instead that neural circuits continue to “re-develop” with experience in adulthood.

**Figure 1:**
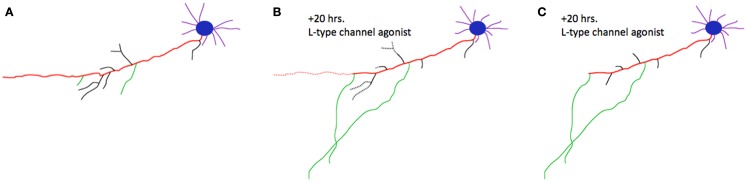
**Competitive axon remodeling accelerated by an L-type calcium channel agonist**. **(A)** Tracing of a cortical neuron grown in dissociated tissue culture. The primary axon is shown in red; branches are shown in black, or green for emphasis. Blue, cell body; purple, dendrites. **(B)** After 20 h in a L-type calcium channel agonist, the primary axon, and many branches dramatically retracted (retractions shown with dotted lines). The green branches were favored for extension in this competitive process; dendrites were largely unaffected. **(C)** Final morphology of the neuron after 20 h. Figure adapted from (2).

Importantly, gene expression data from humans (Human Brain Transcriptome) suggest that the two L-type calcium channel-associated genes identified by the Psychiatric Genomics Consortium (CACNA1C and CACNB2) (1) are broadly expressed in the nervous system. Furthermore, expression of these genes is upregulated between embryonic weeks 13–19, when long-distance axonal tracts such as the corpus callosum are being established (9). Unlike some developmental genes, the expression of these two genes remains high through adulthood. This places these genes at the right times and in a sufficiently broad distribution of brain areas to influence competitive axon remodeling in each of these psychiatric disorders.

Continued axon remodeling throughout life provides a conceptual framework of neurodevelopment (and the slower neural re-development in adulthood) that could go awry during psychiatric illness. As with any proposed mechanism relating the molecular genetics with the observed clinical outcomes, empirical validation, or refutation is required. Validation could yield important alternative signaling pathways that influence competitive axon growth (10). In addition, this type of investigation is more likely to elucidate the biological underpinnings of disease, which the National Institute of Mental Health recently endorsed in favor of symptom-based diagnosis used in the Diagnostic and Statistical Manual of Mental Health. Although it is not currently clear that a single common mechanism links L-type channel mutations to the associated disorders, pursuit of this kind of fundamental link is worthwhile for the conceptual advances and therapeutic targets that might be discovered if true.

## References

[1] Cross-Disorder Group of the Psychiatric Genomics Consortium Smoller JW Craddock N Kendler K Lee PH Neale BM Identification of risk loci with shared effects on five major psychiatric disorders: a genome-wide analysis. Lancet (2013) 381(9875):1371–9 10.1016/S0140-6736(12)62129-1 23453885 PMC3714010

[2] Hutchins BI Kalil K Differential outgrowth of axons and their branches is regulated by localized calcium transients. J Neurosci (2008) 28(1):143–53 10.1523/JNEUROSCI.4548-07.2008 18171932 PMC2474798

[3] Lee H Lee D Park CH Ho WK Lee SH GABA mediates the network activity-dependent facilitation of axonal outgrowth from the newborn granule cells in the early postnatal rat hippocampus. Eur J Neurosci (2012) 36(6):2743–52 10.1111/j.1460-9568.2012.08192.x 22780325

[4] Singh KK Miller FD Activity regulates positive and negative neurotrophin-derived signals to determine axon competition. Neuron (2005) 45(6):837–45 10.1016/j.neuron.2005.01.049 15797546

[5] De Paola V Holtmaat A Knott G Song S Wilbrecht L Caroni P Cell type-specific structural plasticity of axonal branches and boutons in the adult neocortex. Neuron (2006) 49(6):861–75 10.1016/j.neuron.2006.02.017 16543134

[6] Stettler DD Yamahachi H Li W Denk W Gilbert CD Axons and synaptic boutons are highly dynamic in adult visual cortex. Neuron (2006) 49(6):877–87 10.1016/j.neuron.2006.02.018 16543135

[7] Gilbert CD Li W Adult visual cortical plasticity. Neuron (2012) 75(2):250–64 10.1016/j.neuron.2012.06.030 22841310 PMC3408614

[8] Yamahachi H Marik SA McManus JN Denk W Gilbert CD Rapid axonal sprouting and pruning accompany functional reorganization in primary visual cortex. Neuron (2009) 64(5):719–29 10.1016/j.neuron.2009.11.026 20005827 PMC2818836

[9] Ren T Anderson A Shen WB Huang H Plachez C Zhang J Imaging, anatomical, and molecular analysis of callosal formation in the developing human fetal brain. Anat Rec (2006) 288A:191–204 10.1002/ar.a.20282 16411247

[10] Shelly M Lim BK Cancedda L Heilshorn SC Gao H Poo MM Local and long-range reciprocal regulation of cAMP and cGMP in axon/dendrite formation. Science (2010) 327(5965):547–52 10.1126/science.1179735 20110498

